# Analyzing Public Interest in Osteoarthritis and Its Minimally Invasive Treatments: A Google Trends Analysis

**DOI:** 10.7759/cureus.47021

**Published:** 2023-10-14

**Authors:** Cigdem Cinar

**Affiliations:** 1 Department of Physical Therapy and Rehabilitation, Biruni University, Istanbul, TUR

**Keywords:** osteoarthritis, minimal invasive treatment, joint pain, joint disease, google trends

## Abstract

Introduction

In recent years, online search engines have become a source of information about medical issues. We aimed to evaluate the public's interest in osteoarthritis and minimally invasive treatments of osteoarthritis in the last 10 years using Google Trends (GT).

Methods

Interventional physiatrist analyzed 14 selected terms (joint pain, joint pain treatment, joint cartilage damage, narrowing of the joint, osteoarthrosis, osteoarthritis, intra-articular injection, intra-articular steroid, intra-articular hyaluronic acid, intra-articular plasma rich platelet (PRP), essential oil for joint pain, joint pain supplements, home remedies for joint pain, and stem cell for joint) related with osteoarthritis and minimally invasive treatments of osteoarthritis in physical medicine and rehabilitation discipline. All keywords were searched in the GT application using the 'all categories,' 'web search,' and 'worldwide' filters. The last 10 years have been divided into two equal parts, each spanning five years (from January 1, 2013 to December 31, 2017, and January 1, 2018 to December 31, 2022). Public interest in the 14 keywords mentioned above was recorded for these two periods, and the GT for all 14 keywords were compared across the two five-year periods.

Results

Searching rates for the terms 'joint pain,' 'joint pain treatment,' 'joint cartilage damage,' 'narrowing of the joint,' 'osteoarthritis,' 'intra-articular injection,' 'intra-articular PRP,' and 'joint pain supplements' have increased significantly in the last five years (p= 0.001, p= 0.001, p= 0.005, p= 0.001, p= 0.001, p= 0.004, p= 0.001, and p= 0.001, respectively). The average Google Trends (GT) score for all terms was 40 between January 1, 2013, and December 31, 2017, and the average GT score for all terms was 48 between January 1, 2018, and December 31, 2022 (p= 0.001).

Conclusion

The present study stated that public interest in osteoarthritis and minimally invasive treatments for osteoarthritis has increased significantly in the last five years. Study outcomes demonstrated that public attention to 'joint pain,' 'joint treatment,' 'joint cartilage damage,' 'narrowing of the joint,' 'osteoarthritis,' 'intra-articular injection,' 'intra-articular PRP,' and 'joint pain supplements' has also significantly increased in the last five years.

## Introduction

Osteoarthritis is a degenerative joint disease characterized by cell damage, inflammatory processes, and, ultimately, extracellular damage after micro-injury or macro-injury [1]. Previous studies have found that the prevalence of osteoarthritis is growing due to increasingly sedentary lifestyles, rising obesity rates, and longer life expectancy. The global population is aging, and the health and, at the same time, the economic burden of osteoarthritis is increasing [2]. The public interest is also growing in osteoarthritis. In order to help researchers discover the needed information from more sources and more rapidly, the Google search engine became the most popular among the other search engines. Wallace IJ et al. state that almost one in every five people over 45 years of age and more than half of those older than 75 years have osteoarthritis [3]. Although osteoarthritis is most common in the knee, it can develop in any of the body's mobile joints, including the hips, wrists, ankles, fingers, and spine. Previous investigations have determined that osteoarthritis not only increases hospital admissions and leads to greater drug use but also results in workforce losses and negatively impacts social life [4]. Concurrently, numerous internet resources allow patients to easily and cost-effectively access information about the condition [5,6].

Search engines emerged to facilitate the investigations of people seeking information on the internet, enabling them to swiftly find desired information from numerous sources. Although numerous search engines are available today, Google (Google Inc., California, USA) is the predominant choice, accounting for 90% of all searches [7]. The Google Trends (GT) application displays the frequency of a search term's use in Google over a specified period, segmented by region and language [8]. On the GT website (https://trends.google.com), users can find the results for any word searched in the Google search engine. Teng Y et al. show that GT successfully predicted the course of the Zika virus pandemic [9]. In another study, Sevgili E and Baytaroglu C used GT to analyze public attention to cardiac diseases during the COVID-19 pandemic, finding that Google searches for cardiac diseases decreased significantly during that time [10].

Although many studies have used GT to identify the public's interest in various diseases, no research has analyzed public attention to osteoarthritis or minimally invasive treatments for the condition. The present study used GT to evaluate the public's interest in osteoarthritis and minimally invasive treatments for osteoarthritis over the past 10 years.

## Materials and methods

The present study was conducted between August 1, 2023 and August 20, 2023. An interventional physiatrist analyzed 14 selected terms related to osteoarthritis and minimally invasive treatments for osteoarthritis in the disciplines of physical medicine and rehabilitation (joint pain, joint pain treatment, joint cartilage damage, narrowing of the joint, osteoarthrosis, osteoarthritis, intra-articular injection, intra-articular steroid, intra-articular hyaluronic acid, intra-articular plasma rich platelet (PRP), essential oil for joint pain, joint pain supplements, home remedies for joint pain, and stem cell for joint). The keywords were selected according to the Osteoarthritis Clinical Practice Guidelines of the American College of Rheumatology. All the keywords were searched in the GT application using the "All categories," "Web search," and "Worldwide" filters.

Google Trends

**Figure 1:**
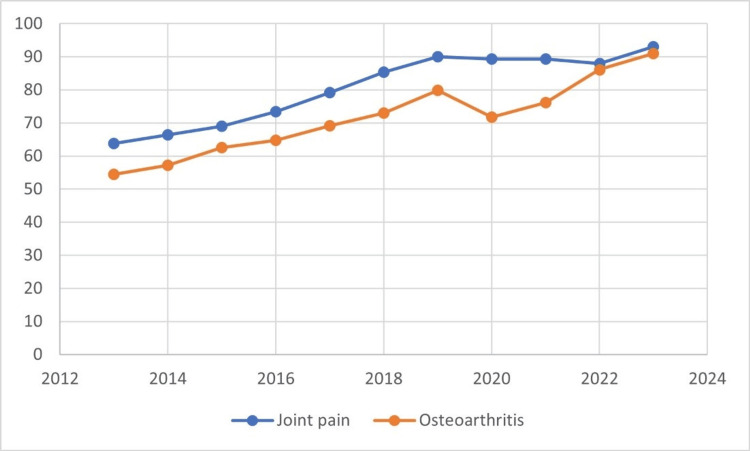
Changes in the terms 'joint pain' and 'osteoarthritis' by years in the Google Trends data.

GT provides statistical information about the frequency with which a word is searched in Google's search engine relative to similar words over a specified time period. This feature of GT is pivotal in establishing a website or enabling advertisers to optimize their reach when crafting advertisements. The GT score for any term ranges from 0 to 100, where 0 indicates the lowest rate of public interest and 100 represents the highest [11]. To identify public attention to osteoarthritis and minimally invasive treatments for the condition in the past 10 years using GT, the past 10 years were divided into two equal parts of five years each (from January 1, 2013 to December 31 2017 and January 1, 2018 to December 31 2022). Public interest in the previously described 14 keywords was identified in these two periods, and then the trends of all 14 keywords were compared between the two five-year periods. The research also analyzed the distribution of searches for the terms joint pain and osteoarthritis by year and country. It evaluated changes in the terms joint pain and osteoarthritis by year in the GT data (Figure 1).

Ethics committee approval was not obtained because the study did not use patient data.

Statistical analysis

The analysis was performed using IBM SPSS version 27 (IBM Corp., Armonk, NY, USA). Relative search volume (RSV) data from January 1, 2013 to December 31, 2017 and January 1, 2018 to December 31, 2022 were pooled, and the RSV means were calculated separately. The normality of the variables' distribution was analyzed with the Kolmogorov-Smirnov test, and the comparison of RSV between the two periods was done using the Mann-Whitney U test. The data were analyzed at a 95% confidence level, and a p-value of less than 0.05 was accepted as statistically significant.

## Results

The comparison of GT data between the two five-year periods is summarized in Table 1. The search rates for the terms joint pain, joint pain treatment, joint cartilage damage, narrowing of the joint, osteoarthritis, intra-articular injection, intra-articular PRP, and joint pain supplements have increased to a statistically significant degree in the past five years (p=0.001, p=0.001, p=0.005, p=0.001, p=0.001, p=0.004, p=0.001, and p=0.001, respectively). By contrast, the terms osteoarthrosis and home remedies for joint pain have been searched less frequently in the past five years (both p=0.001). Lastly, search rates for the terms intra-articular steroid, intra-articular hyaluronic acid, essential oil for joint pain, and stem cell for joint were compared according to years and were found to be similar between the two periods (p=0.600, p=0.645, p=0.436, and p=0.654, respectively). The average GT score of all the terms was 40 in the years 2013-2017 and 48 in the years 2018-2022 (p=0.001).

**Table 1:** Distribution of search frequency for terms by years. * median (IQR). PRP: Plasma rich platelet.

Terms	From January 1, 2013 to January 31, 2017	From January 1, 2018 to December 31, 2022	P-value
Joint pain*	70 (66-78)	90 (86-92)	0.001
Joint pain treatment*	63 (58-73)	81 (77-87)	0.001
Joint cartilage damage*	35 (25-47)	45 (31-59)	0.005
Narrowing of the joint*	33 (23-43)	55 (43-66)	0.001
Osteoarthrosis*	66 (53-82)	49 (45-54)	0.001
Osteoarthritis*	64 (58-69)	79 (74-85)	0.001
Intra-articular injection*	61 (50-72)	67 (57-80)	0.004
Intra-articular steroid*	19 (0-41)	21 (12-32)	0.6
Intra-articular hyaluronic acid*	20 (0-34)	17 (0-28)	0.645
Intra-articular PRP*	21 (0-42)	44 (18-62)	0.001
Essential oil for joint pain*	23 (10-45)	25 (12-43)	0.436
Joint pain supplements*	49 (44-53)	73 (68-82)	0.001
Home remedies for joint pain*	21 (4-36)	14 (0-21)	0.001
Stem cell for joint*	16 (2-24)	18 (4-28)	0.654
Total*	40 (33-49)	48 (41-56)	0.001

**Figure 2:**
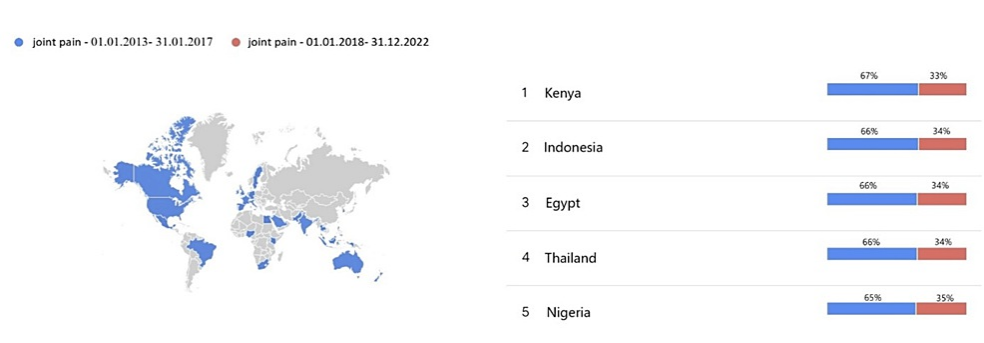
Distribution of searches for the term 'joint pain' by years and countries.

**Figure 3:**
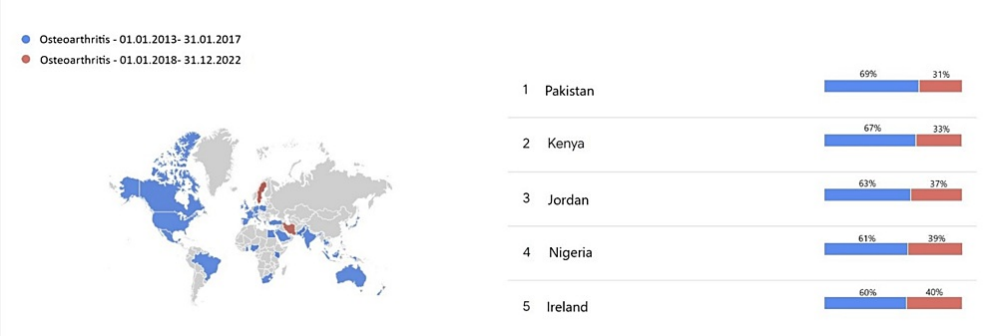
Distribution of searches for the term ‘osteoarthritis’ by years and countries.

The term joint pain was searched more in all countries in the past five years than in the years 2013-2017. The most pronounced difference between the search rates of the past five years and the previous five years was found in Kenya, Indonesia, Egypt, Thailand, and Nigeria (Figure 2a). The term osteoarthritis was searched more in the past five years than in 2013-2017 in many countries, but the search rates have decreased in the past five years in Sweden and Iran (Figure 2b). The search data for the terms 'joint pain' and 'osteoarthritis' over the past 10 years are presented in Figure 3. Both terms have been searched more frequently in the past five years than in the preceding five-year period, and the search volumes for these two terms exhibit a cumulative increase from 2013 to 2023.

## Discussion

Patients and their relatives have widely utilized internet-based sources to assess their symptoms and garner additional information about their diseases, treatment outcomes, and disease prevention strategies [12]. The prevalence of joint diseases has increased appreciably worldwide for reasons such as obesity, sedentary lifestyles, and prolonged life expectancy, making osteoarthritis a notable health concern [13]. We believe that clarifying the terms related to osteoarthritis that are searched more frequently on Google will increase awareness of the condition and predict which minimally invasive treatment methods for osteoarthritis attract the public's attention. Thus, we conducted a study using GT to understand public interest in osteoarthritis and its minimally invasive treatments over the past 10 years. The results show public interest in osteoarthritis and minimally invasive treatments has increased significantly in the past five years. In addition, the study outcomes reveal that public interest in joint pain, joint pain treatment, joint cartilage damage, narrowing of the joint, osteoarthritis, intra-articular injection, intra-articular PRP, and joint pain supplements has significantly increased in the past five years. Increasing awareness of a disease can enable patients to approach health systems in a shorter time and allow the disease to be treated before it reaches the irreversible stage. Plackett R et al. reviewed 23 studies on social media use and the early diagnosis of cancer and found that social media played a pivotal role in cancer screening and the early diagnosis of patients [14]. Furthermore, Mandrola J and Futyma P claim that the period between the publication of information as a scientific article and its introduction into clinical practice is over 15 years. However, the use of social media has reduced this period [15]. Additionally, Nuti SV et al. show that GT scores for numerous diseases have increased significantly in recent years [16]. The present study found that the terms joint pain, joint pain treatment, articular cartilage damage, and osteoarthritis have been searched significantly more in the past five years. Our findings suggest that producing content with the aforementioned words will enable patients to access the right information more quickly and easily. Among the most popular topics are pain treatment in joint disorders and intra-articular treatments. Urits I et al. note that achieving pain relief is crucial in joint disease, and the authors emphasize that pain supplements were the first treatment options for pain relief [17]. McAlindon TE et al. also indicate the importance of intra-articular injections in treating joint pain and joint diseases [18]. In their GT analysis, Strotman PK et al. found increasing public attention to intra-articular stem cell injection for hip and knee osteoarthritis [19]. In another study, Brinkman JC et al. analyzed public interest in PRP injections (albeit only in the shoulder), concluding that public interest in shoulder PRP injections has increased steadily over 10 years [20]. The present study's findings demonstrate that public interest in intra-articular injection, intra-articular PRP, and joint pain supplements has significantly increased in the past five-year period.

Limitations of the study

Although this study is the first to investigate the public's interest in osteoarthritis and minimally invasive methods of osteoarthritis treatment, the research was conducted using only Google search engine results, representing a limitation of the study. Numerous web search engines are available, but almost 9 out of 10 people prefer Google when using a search engine. Furthermore, the terms were searched only in English, but many languages other than English can be used on Google. However, English is the most common language used in the search machine. Finally, we adopted 14 terms in this study, but people can use different terms to search for information about osteoarthritis and minimally invasive treatments for osteoarthritis through Google.

## Conclusions

The present study found that public interest in osteoarthritis and its minimally invasive treatment has increased significantly in the past five years. In addition, the study outcomes demonstrated that public attention to joint pain, joint pain treatment, joint cartilage damage, narrowing of the joint, osteoarthritis, intra-articular injection, intra-articular PRP, and joint pain supplements significantly increased in the past five years. Creating web content about osteoarthritis and its minimally invasive treatments, utilizing keywords frequently searched on Google, will positively impact public joint health.
